# Social cognition in individuals born preterm

**DOI:** 10.1038/s41598-021-93709-4

**Published:** 2021-07-14

**Authors:** Marina A. Pavlova, Jessica Galli, Federica Zanetti, Federica Pagani, Serena Micheletti, Andrea Rossi, Alexander N. Sokolov, Andreas J. Fallgatter, Elisa M. Fazzi

**Affiliations:** 1 Department of Psychiatry and Psychotherapy Tübingen Center for Mental Health (TüCMH), Medical School and University Hospital, Eberhard Karls University of Tübingen, Calwerstr. 14, 72076 Tübingen, Germany; 2 Department of Clinical and Experimental Sciences, University of Brescia, Brescia, Italy; 3 Unit of Child and Adolescent Neurology and Psychiatry, ASST Spedali Civili di Brescia, Brescia, Italy

**Keywords:** Neuroscience, Social neuroscience

## Abstract

Faces hold a substantial value for effective social interactions and sharing. Covering faces with masks, due to COVID-19 regulations, may lead to difficulties in using social signals, in particular, in individuals with neurodevelopmental conditions. Daily-life social participation of individuals who were born preterm is of immense importance for their quality of life. Here we examined face tuning in individuals (aged 12.79 ± 1.89 years) who were born preterm and exhibited signs of periventricular leukomalacia (PVL), a dominant form of brain injury in preterm birth survivors. For assessing the face sensitivity in this population, we implemented a recently developed experimental tool, a set of Face-n-Food images bordering on the style of Giuseppe Arcimboldo. The key benefit of these images is that single components do not trigger face processing. Although a coarse face schema is thought to be hardwired in the brain, former preterms exhibit substantial shortages in the face tuning not only compared with typically developing controls but also with individuals with autistic spectrum disorders. The lack of correlations between the face sensitivity and other cognitive abilities indicates that these deficits are domain-specific. This underscores impact of preterm birth sequelae for social functioning at large. Comparison of the findings with data in individuals with other neurodevelopmental and neuropsychiatric conditions provides novel insights into the origins of deficient face processing.

## Introduction

Preterm birth is defined as any birth before 37 completed weeks of gestation, or fewer than 259 days since the first day of the woman’s last menstrual period^1^. It affects approximately 11% of births worldwide^2,3^. As a consequence of the progress in pre- and neonatal intensive care and advances in medical knowledge and technology, the survival rate of preterm infants is continuously increasing^3,4^. Quality of survival, therefore, has become a major concern with clear social and clinical relevance^5,6^. Daily-life social participation of preterm born (PB) individuals is of immense value for their quality of life. Low social competence, even more prevailing than other behavioural problems, may lead to social withdrawal^7–12^. Former preterms are reported to express poorer social knowledge and reasoning abilities than their term-born peers^13^.

**Figure 1:**
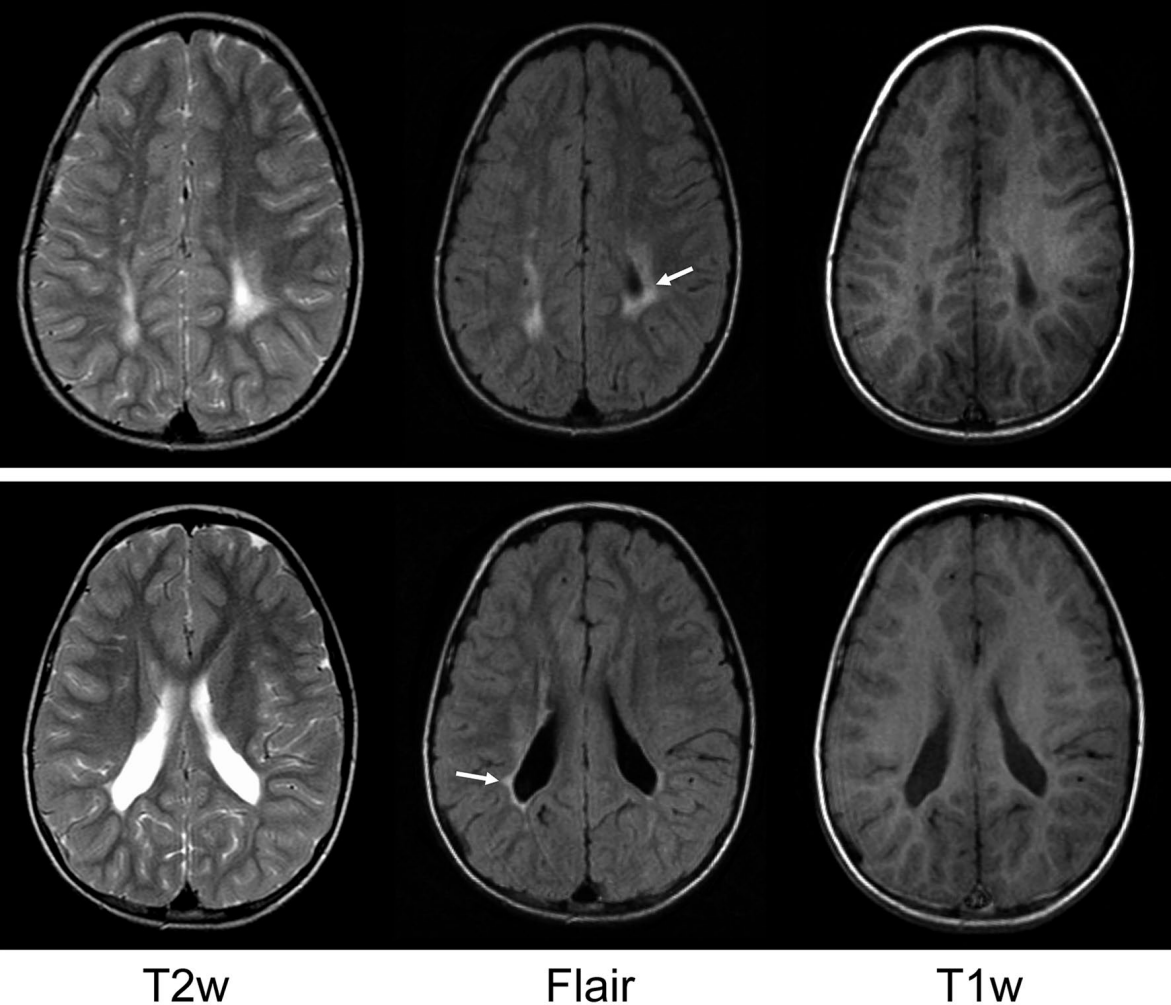
Periventricular leukomalacia (PVL) as depicted by MRI at a later stage after completed myelination in a 6-year-old male born at 32 weeks of gestation. Axial T2-weighted (T2w), FLAIR and T1-weighted (T1w) images at the level of the centrum semiovale cutting the upper part of the ventricles (top row) and at the level of the lateral ventricles (bottom row) show the characteristic gliosis as hyperintense high signal on the T2-weighted image and hypointense low signal on the T1-weighted image. The gliosis is best seen on FLAIR images, where the periventricular hyperintensities contrast with the hypointense CSF signal. The top row illustrates gliosis, and the bottom row additional tissue loss (mild ventricular dilatation with irregularly extended borders). From Ref.^5^.

Faces and body language are two sources of non-verbal information most essential for efficient daily-life mutual interactions^14–24^. As argued earlier^5^, for a long time, both components were under-investigated in survivors of preterm birth. The visual sensitivity to body motion (represented by a set of light dots on the main joints of the invisible actor’s body) emerges early in development^25–28^. Already 3-day-old newborns visually prefer point-light human locomotion^29^. Even newly hatched chicks (*Gallus gallus*) are likely to be predisposed to biological motion of other species as well as to animacy in general^30–33^, though such predispositions are impaired in newborns at high risk of autism^34,35^ and in young autistic children^36^. The capacity for extracting information from body motion appears to be intrinsically tied with social cognitive abilities such as understanding of drives and emotions of others^17^. For instance, inferring affect from point-light locomotion and performance on the Reading the Mind in the Eyes Test (RMET) are strongly tangled in adult females^37^. Extracting social information from body motion is reported to be aberrant in survivors of preterm birth (for review, see^5^). However, the origins of these deficits are difficult to evaluate, since samples often include individuals with different aetiology of brain lesions, and the data are collapsed across PB individuals with and without brain injury (e.g., periventricular leukomalacia, PVL). PVL is a dominant form of brain injury in survivors of preterm birth^5^ (Fig. 1). Being a result of necrosis of fibres around the lateral ventricles in the peritrigonal area, PVL is characterized by periventricular gliosis in the white matter with or without tissue loss and secondary ventricular dilatation affecting connectivity of subcortical structures with cortical areas, in particular, the posterior thalamic radiation, and cortico-cortical connectivity^5,38–40^. Alterations are also reported in the integrity and volume of inferior fronto-occipital fasciculus, the superior longitudinal fasciculus, and frontal aslant tract^41^. PVL represents a bilateral pattern of lesions that constrains the compensatory capability of the brain, as it affects both hemispheres and, therefore, limits the developing brain for reorganization to preserve functions at risk^5^.

Pioneering work in this domain indicates that PB adolescents with signs of PVL exhibit compromised ability for visual body motion processing as compared not only with term-born peers, but also with preterms without structural brain abnormalities detectable on magnetic resonance imaging, MRI, scans, and the severity of this impairment is related to the volumetric extent of parieto-occipital PVL^42,43^. Shortages in body motion perception are unrelated to severity of motor disability, leg-dominated cerebral palsy (in other words, the sensitivity to body motion is not affected by the observers’ early restrictions in their own locomotion^42^, gestation age, and birth weight^44^). In former preterms aged 8–11 years with extremely low birthweight (less than 1500 g) autistic traits are reported to be positively related to the ability to reveal identity of a point-light performer (i.e., individuals with more pronounced autistic traits are more sensitive to identity of an actor defined through body motion that appears paradoxical), whereas autistic traits are negatively correlated with a capability to detect a camouflaged walking person in both typically developing (TD) and PB children^45^. This same cohort of children is less capable of extracting social attribution from the Heider-and-Simmel-like films with moving geometric shapes (a valuable tool for social competence examination^5,17,46^): they under-attribute intentionality and mental states to the shapes^47^. These children also turn to be less proficient in decoding non-verbal social signals from persons engaged in naturalistic dynamic social interactions in the Child and Adolescent Social Perception test (CASP^48^), and their difficulties are related to autistic traits^49^. Impairments on all these tasks suggest that PB individuals experience generalized hitches in processing of social information revealed through motion. Yet PB adolescents aged 13–16 years with PVL are substantially impaired also on the event arrangement task requiring understanding of others (that is not based on motion characteristics), whereas their PB peers with normal MRI scans perform much like term-born children. This deficit is related to the PVL extent in the right temporal brain area known to be heavily engaged in social cognition^50^.

Along with body language, faces hold an exceptional value for a wide range of social functioning^16,21^. Only a very few indications of deficits in face processing associated with prematurity were available till last decade^51,52^. Deficient face recognition (as assessed by the Facial Memory subtest from the Test of Memory and Learning, TOMAL^53^) is reported in 18% of 22 premature children aged 6–15 years with signs of (mostly) moderate parieto-occipital PVL identifiable on MRI scans^54^. Recent work reveals alterations in face processing as sequelae of preterm birth. PB infants examined during the first 48 h of life do not express visual preference for faces over scrambled faces as their term-born peers do^55^. Functional near-infrared spectroscopy (fNIRS) indicates that PB infants aged 6–10 months show smaller hemodynamic response in the right frontotemporal areas while watching their own mother’s faces as compared to unknown faces, whereas in term-born infants, mothers’ faces elicit greater hemodynamic responses than unknown faces^56^. Former preterms aged 6–8 months demonstrate atypical eye-gaze behaviour in regard to faces and social content at large: they look shorter at eyes and a face presented among non-face distractors than their term-born peers^57^. If small for gestational age, former preterms 5–15 years old perform worse on the Facial Memory subtest from the TOMAL^53^ showing poorer face recognition skills^58^. However, former preterms aged 19–20 years display intact skills in terms of accuracy (albeit slower responses) for inferring emotions from dynamic faces, with some difficulties in recognizing anger at low intensity^59^. Adults (26–36 years old) who were born preterm with extremely low birthweight (< 1000 g) possess lower ability for discrimination between individual faces, whether human or monkey^60^, indicating that deficits in face processing are long-lasting and persist into adulthood.

**Figure 2:**
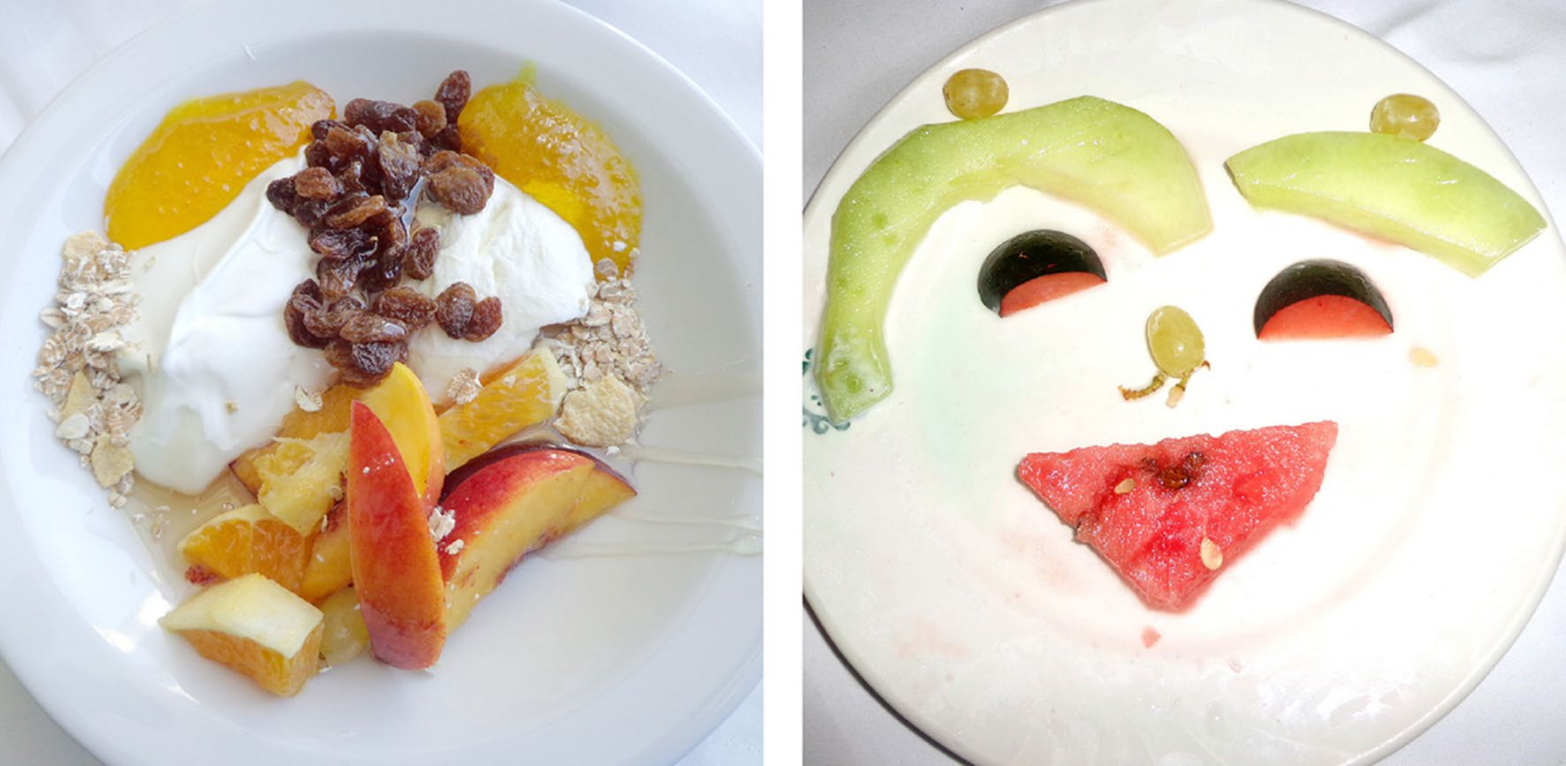
Examples of Face-n-Food images. The least resembling face (left panel) and most resembling face (right panel) images from the Face-n-Food task (from Ref.^61^; the Creative Commons Attribution [CC BY] license).

The present study was directed at investigation of face tuning in PB adolescents who suffer PVL. For investigation of face tuning, we applied a recently developed tool, a set of images comprising food ingredients such as fruits and vegetables^61–68^. The Face-n-Food images to some extent border on the style of Giuseppe Arcimboldo, a genius Italian painter known for his imaginative portraits composed of fruits, vegetables, and even roasted meat (Fig. 2). The primary advantage of these images is that single components do not trigger face processing. In other words, on the Face-n-Food task, face tuning occurs spontaneously without being explicitly cued by face elements such as eyes or mouth. For seeing a face in these images, one has to perceive an image as a Gestalt (a German word that defines a configuration of elements unified into a whole in such a way that its overall properties cannot be identified from a simple sum of its parts). The other advantage of the task is the usage of unfamiliar images that is of value for research in clinical populations^69^. Here we intended to clarify (i) whether former preterms exhibiting PVL express aberrant face tuning; and (ii) whether possible shortfalls in the face sensitivity are related to other visuo-perceptual abilities or rather represent a specific deficit. For assessing visuospatial and cognitive abilities, additional tasks were administered to PB individuals. All of them represent well-established tools for neuropsychological assessment^54,70^ (for description, see “Neuropsychological assessment” section in “Methods” section).

## Results

The Face-n-Food images were presented to all participants one by one in a predetermined order from the least to most recognizable as a face^61^. Similar to previous work in TD individuals and in a range of neurodevelopmental and psychiatric conditions^61–68^, PB individuals described a food-plate image either in terms of food (non-face response, 0) or as a face (face response, 1). Two out of 14 PB patients completely failed on the Face-n-Food task: they did not have spontaneous face impressions even from the most face-resembling images. PB individuals reported seeing a face for the first time on average only on image 7.33 ± 1.37 (mean ± SD; Mdn, 7, 95% CI, 6.56 to 8.11). TD controls gave the first face response on average on 3.56 ± 1.59 image (Mdn, 3.5; 95% CI 2.78 to 4.34), and all of them perceived images most resembling faces as a face. Thresholds for the face tuning (a median image number, on which a face response was given for the first time) were much higher in PB patients than in TD controls (Steel–Dwass test, *z* = 4.426, *p* < 0.0001; effect size, Cohen’s *d* = 2.744). Comparison with earlier data in autistic children of the same age and cultural background^64^ reveals that the face sensitivity in PB individuals is also lower than in persons with ASD (Steel–Dwass test, *z* = 2.375, *p* < 0.046; effect size, *d* = 0.962).

**Figure 3:**
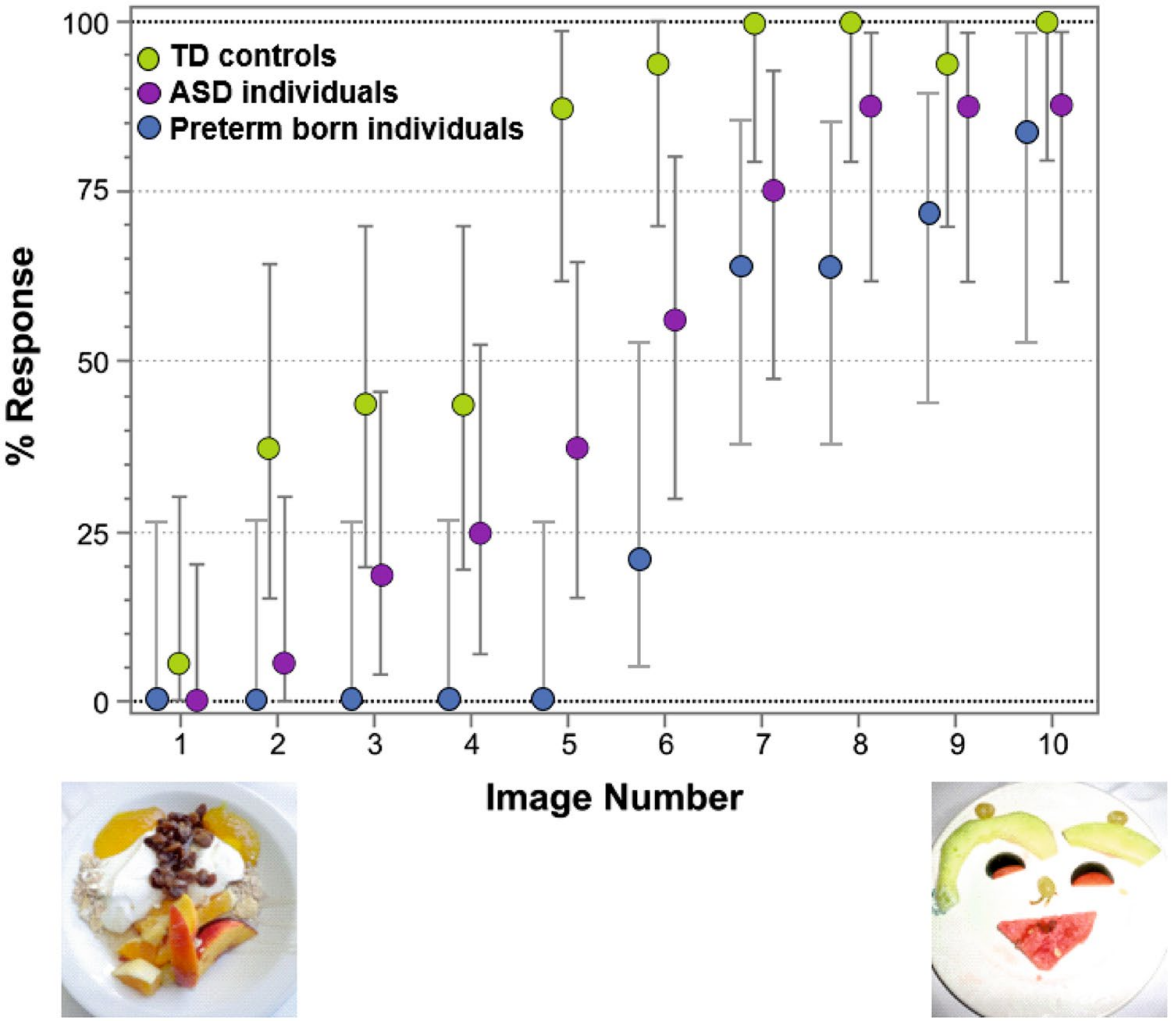
Percentage of face responses for each Face-n-Food image in TD controls (green), former preterms with PVL (blue), and patients with autistic spectrum disorders (ASD, violet). The image number reflects its face resemblance (1, the least recognizable as a face, through 10, the most resembling a face images from the Face-n-Food task; see Ref.^61^; the Creative Commons Attribution [CC BY] license). Vertical bars represent 95% confidence interval, CI. The data for individuals with ASD had been reported earlier (see Ref.^64^), and provided here for comparison solely.

Figure 3 represents the percentage of face responses for each Face-n-Food image separately for former preterms and TD controls. Inspection of this Figure shows that former PB adolescents not only much later had a face impression, but gave overall much fewer face responses. As indicated by multiple stepwise nominal logistic regression analysis, the effect of group (TD vs. PB) is highly significant (*χ*^2^(1) = 96.01, *p* < 0.0001; effect size, *r* = 1.789). Remarkably, for the first five images that are less face resembling, all PB individuals provided no face responses at all. Starting from the image 5, TD participants reached very fast the ceiling level of performance. By contrast, even on the images strongly resembling a face (7 through 9), PB individuals attained about 70% of face responses, and only the most recognizable image 10 elicited 85.71% face responses.

Figure 3 enables comparison of the data in PB individuals not only with TD controls, but also with earlier data in individuals with autistic spectrum disorders, ASD^64^. Multiple stepwise nominal logistic regression analysis indicates that all groups of participants significantly differ from one another (*χ*^2^(2) = 78.37, *p* < 0.0001). Most eye-catching finding is that the performance level of PB patients on the Face-n-Food task is substantially lower not only as compared with TD controls, but also with ASD individuals (*χ*^2^(1) = 18.38, *p* < 0.0001; effect size, *r* = 0.783; with no difference in the general intelligence quotient, GIQ scores between PB and ASD persons: *t*(28) = 1.124, n.s.).

No correlation was found between performance of PB individuals on the Face-n-Food task (face response rate) and the GIQ (Spearman’s rho, *ρ* = 0.057, *p* = 0.85, two-tailed, n.s.). There was also a lack of correlation between face response rate and performance on the visual-perceptual tests: the Block Construction (*ρ* = 0.236, *p* = 0.416, n.s.), Street Completion (*ρ* = 0.213, *p* = 0.465, n.s.), VMIG (*ρ* = 0.397, *p* = 0.16, n.s.), Unusual Perspective (*ρ* =  − 0.011, *p* = 0.969, n.s.), Unusual Lighting (*ρ* = − 0.134, *p* = 0.648, n.s.), and IF tests (*ρ* = 0.295, *p* = 0.306, n.s.). As expected, no correlation was found between the face tuning and tests assessing semantic categorization: Matching by Class (*ρ* = 0.077, *p* = 0.795, n.s) and Matching by Function (*ρ* =  − 0.077, *p* = 0.794, n.s.). This indicates that impaired performance on the Face-n-Food task in PB individuals originates from specific deficits in the face sensitivity rather than from other visual-perceptual or cognitive disabilities.

## Discussion

By applying a novel tool for the face sensitivity examination, a recently developed Face-n-Food task^61–68^, we assessed the face tuning in individuals who were born prematurely and suffer PVL, the dominant form of brain injury in prematurity^5,54^. PVL represents a bilateral pattern of white matter lesions equally affecting both hemispheres that leaves the brain without sufficient potential for functional reorganization. The outcome indicates that in former survivors of preterm birth with PVL, the face sensitivity is substantially lower than in term-born TD peers. In PB individuals, the face sensitivity in face-like non-face images is not related to visual-perceptual organization and other cognitive abilities assessed in the course of neuropsychological examination and, therefore, the lower performance on the Face-n-Food task appears to stem from deficits in the face tuning per se, or strictly speaking, in the sensitivity to a coarse face schema.

### Face tuning in prematurity and autism

Evaluation of the outcome in PB individuals within the framework of earlier data obtained in a sample of individuals with ASD^64^ of similar age, cultural background (known to affect the face sensitivity^66^), gender (both samples are prevailed by males) and general IQ, reveals that PB individuals with PVL express a lower sensitivity to a coarse face schema in the Face-n-Food images as compared not only with TD term-born controls, but also with their peers with ASD. Although in both clinical samples, a number of patients who completely failed on the task is comparable (i.e., two out of 16 in ASD and two out of 14 in PB patients did not spontaneously recognize even the most face resembling image 10), the face recognition dynamics is different in these groups. Face recognition of the first five-six images less resembling a face is remarkably poor in PB with PVL (Fig. 3). Only starting from image 7, PB individuals reliably recognize images as a face, reaching 85.7% recognition for the most face resembling image 10. By contrast, face recognition of ASD individuals steadily improves resulting in 87.5% face responses for each of the most recognizable images 8–10. As ASD individuals are well-known to experience difficulties in several aspects of social cognition^17,18,35,36,71^, this discrepancy in the face sensitivity between ASD and PB patients (with substantially lower face tuning of former preterms) appears arresting. Survivors of preterm birth are reported to express autistic traits as well as difficulties in social functioning such as social withdrawal, incompetence, and communication problems^72^, or they can be also positively screened for ASD^73–75^. However, this cannot provide a satisfactory explanation for such a poor outcome in PB individuals. As establishing binding between even a couple of elements resembling eyes and mouth, i.e., building a coarse face scheme, is already sufficient for perception of non-face images as a face^76^, it appears stunning that PB patients are so deeply impaired on the task. Furthermore, a kind of predisposition for a coarse face schema (such as two eyes above a mouth) is believed to occur already very early in lifespan^77^: foetuses in the third trimester of pregnancy^78^, human infants^79–82^, and children aged 5–6 years^83^ are reported to demonstrate a visual preference for face-like stimuli over similar images. Furthermore, even newly hatched domestic chicks (*Gallus gallus*) exhibit preference to a coarse face schema^84–86^, while non-human primates such as the rhesus monkey are likely to share face-detection machinery with humans^87–89^. Yet it is reported that only 7- and 8-month-old human infants (but not infants aged 5-6 months) exhibit visual preference to Archimboldo portraits over the same images presented upside-down^90^ that implies a period of development of the sensitivity to faces in such images.

### Impact of PVL on the face tuning

PVL lesions specifically affecting neural networks underwriting face processing might be primarily responsible for the poor outcome on the Face-n-Food task in former preterms. The periventricular area of the brain contains many interconnecting fibres, damage to which may lead to brain disintegration eliciting a number of deficits in visual perception and social cognition. Converging evidence from neuroimaging, neuropsychological and electrophysiological studies indicate that the subcortical face route, such as colliculo-pulvino-amygdalar pathway^91^, provides a neural substrate for the cortical *social brain* network^87,89,92,93^. Alterations of this pathway along with subcortical-cortical and cortico-cortical connectivity caused by PVL might be decisive for alterations in face processing and social cognition in PB individuals. Comparison of face tuning in PB individuals with and without PVL lesions identifiable on a MRI scan may deliver pivotal arguments for the sources of deficient face tuning. Yet one has to keep in mind that the altered brain connectivity in atypical perinatal conditions, such as Intrauterine Growth Restriction (IUGR) and prematurity itself (in former preterms without brain injury identifiable by brain imaging), have been shown to impact the brain development, affecting network connectivity in the short- and long-term and resulting in the impairment in socio-cognitive potential in childhood and adolescence^94–99^. In addition, fNIRS points to a negative correlation in PB individuals between oxyhemoglobin HbO2 measures during presentations of faces and grey matter volumes in the face-specific brain regions forming the social brain such as the fusiform gyrus and amygdala^56,100^.

Earlier data indicates that PB individuals with PVL exhibit compromised ability for visual processing of body motion as compared not only with term-born peers, but also with former preterms without structural brain abnormalities detectable on MRI scans^43,101^. The same holds true for the visual social cognition (namely, event arrangement) task requiring perception and understanding of others: only former preterms suffering PVL are impaired on this task, and this deficit is related to the lesion extent in the right temporal region^50^. By affecting structural and functional brain connectivity, PVL may lead to disintegration of neural communication inside and outside of the networks supporting social cognition. In line with this assumption, in adolescents with PVL, bursts in the spectral amplitude of the magnetoencephalographic (MEG) cortical response underlying large-scaled neural communication occur later than in TD adolescents or are even completely absent^101^ (see also^102,103^ for evidence on alterations in the topography of the evoked MEG response to body motion with and without a camouflage).

### Specificity of face tuning in prematurity

Previous work revealed substantial (though rather specific for every single neurodevelopmental and neuropsychiatric condition) deficits in the face tuning in different patient populations (for comparative analysis, see^67^). For example, in schizophrenia, the face tuning is substantially impaired, and this deficit is related to deficits in both social cognition and visual perceptual organization^67^, whereas individuals with major depressive disorder do not express any shortfalls in face tuning^68^. Comparison of the present outcome with earlier data in patients with Down syndrome, DS^65^, and Williams syndrome, WS^62^, sheds light on the possible origins of the deficient face tuning in former preterms. By contrast with ASD patients, both WS and DS individuals are well-known for their appetitive drive for social interaction and face fascination. Yet all three samples of patients exhibit poor tuning to coarse face information in face-like non-face images: all of them did not recognize the first five images (less resembling a face) as a face. However, individuals with DS remain on rather low face recognition rate (about 0.3) even for the most recognizable image 10^65^ (Fig. 3) that can originate from some difficulties in abstract/symbolic reasoning, whereas PB individuals as well as those with WS steadily improve in their face recognition reaching 85.7% and 85%, respectively, for the most resembling a face image 10. This analysis suggests that face tuning deficits may be of similar origin in PB and WS individuals. One possible explanation is that difficulties in feature integration and Gestalt perception (perceiving images as a whole) known in both patient groups^5,62,104–107^, underlie deficient face tuning. On the same wavelength, original Arcimboldo hidden-face portraits are judged as being more ambiguous by adult TD individuals with local perceptual style^108,109^. The next step in clarification of the nature of aberrant face tuning in prematurity would be functional brain imaging during perception of face-like non-face images. Such examination, in particular, by means of MEG uncovering even fine-graded alterations in the time course and dynamics of bran activity, helps to identify potential deficiencies in neural communication of the underpinning neural networks. Another valuable approach would be using of multimodal integrative strategies that combine several sources of information (such as structural and functional brain connectivity) in relation to behavioural measures of performance^22,24^.

### Face tuning in prematurity and functional brain imaging

Intact communication inside and outside of the brain networks underlying face tuning is largely unidentified and the findings available are rather controversial^67^. Taking together, the findings demonstrate that (a) topography and time course of the neural circuits underpinning processing of real faces and face-like images are similar; key activation includes the occipital cortices, fusiform face area (FFA), and inferior temporal brain areas^110–112^; and (b) corresponding brain activation is primarily right-hemispheric^113,114^. The right hemispheric dominance is also reported in processing of Arcimboldo-like images yielding greater functional magnetic resonance imaging (fMRI) activation, compared to Renaissance portraits and faces, in the occipito-temporal face-specific network (covering the FFA), bilateral superior and inferior parietal cortices, and the inferior frontal gyrus^109^. The right superior temporal sulcus (STS), a hub of the social brain, distinguishes real faces from face-like images^115^. Electroencephalography, EEG, suggests that already 1- to 4-day-old newborns exhibit activation in the right-lateralized network engaging lateral occipito-temporal and medial parietal areas overlapping with the face-processing circuits in adults^82^. However, fNIRS conducted in 7–8-month-old infants indicates that in response to upright Arcimboldo portraits compared with images of single vegetables used as a baseline, the concentration of oxy-Hb increases in the left (but not right) temporal area, whereas such effect is absent in response to inverted Arcimboldo images in the temporal areas of both hemispheres^90^. The findings in patients with lesions suggest that lesions to the right ocipito-temporal brain areas leave perception of Arcimboldo portraits as faces intact^116,117^, whereas left-hemispheric lesions severely affect face tuning^118^. Individuals with pre-manifest Huntington’s disease (characterized by aberrant social cognition^119^) show a right hemispheric decrease in the N170 component of event related potential, ERP, elicited by the face-like non-face images^120^.

### Sex impact on the face tuning in prematurity

As predominantly male PB patients had been enrolled in the present work, one of the study’s limitations is that it left possible sex differences beyond attention. It is known that males are at a 14–20% higher risk of premature birth^121–123^ and its complications, being more vulnerable to white matter injury^5,124,125^. In the face sensitivity, female superiority has been observed by administering the Face-n-Food task to a homogeneous group of university students^61^. Yet in adult individuals with major depression, no gender differences occur in the face tuning^68^. The female brain is reported to be more responsive to face-like non-face images such as clocks or backpacks eliciting a face impression, with a greater activation in the right STS and Brodmann area 22, though sex differences are absent at earlier stages of face processing^112^. Therefore, the rough face schema appears to be sex-independently hardwired in the typically developing brain. Future work is required to elucidate possible sex differences in the face sensitivity of former preterms both at behavioural and brain levels. However, most likely, sex differences will be camouflaged by a more influential impact of PVL, and, therefore, clarification of this issue appears more plausible in former preterms without signs of severe brain abnormalities.

## Résumé

Although a course face schema is thought to be hardwired in the brain, individuals who were born preterm with signs of brain injury (PVL) exhibit substantial deficits in the face tuning not only compared with typically developing controls but also with individuals with ASD. The lack of associations between the face sensitivity and other cognitive abilities indicates that these deficits most likely stem from alterations in face tuning per se. This underscores impact of preterm birth sequelae for social functioning at large.

## Methods

### Participants

Fourteen adolescents (5 females, 9 males) born preterm were enrolled in the study. They were recruited at the Unit of Child and Adolescent Neurology and Psychiatry of ASST Spedali Civili (Civil Hospital) of Brescia, Italy. PB participants were aged 12.79 ± 1.89 years (mean ± SD; age range, 10 to 16 years). Their gestational age at birth was 30.64 ± 3.13 weeks (ranging from 26 to 36 weeks); 3 of them were born extremely preterm (less than 28 weeks of gestation), 8 were born very preterm (28–32 weeks), and 3 were moderate to late preterm (32–36 weeks). Their birthweight was on average 1710 ± 756 g (median, Mdn, 1473 g, 95% confidence interval, CI, from 1313 to 2106) ranging from 940 to 3300 g. The general IQ (GIQ, Wechsler Intelligence Scale for Children, WISC, adapted to Italian population) of PB participants was on average 92.36 ± 14.17, range of 77 to 126 (6 of them had GIQ higher than 90). All of them had signs of PVL on a structural MRI scan affecting primarily parietoocipital periventricular areas, and neurologically exhibited signs of cerebral palsy in a form of diplegia or tetraplegia. Sixteen TD controls (1 female, 15 males; aged 14.13 ± 2.14; age range, 11 to 17 years; with no age differences with PB group; t(28) = 1.59, two-tailed, n.s.) had been recruited from the local community of Brescia, Italy. The data of this group was previously reported within the framework of the studies on autism and Down syndrome^64,65^. Participants were run individually. All of them had normal or corrected-to-normal vision. None had previous experience with such images and tasks. The study was conducted in accord with the Declaration of Helsinki and was approved by the local Ethics Committee of ASST Spedali Civili (Civil Hospital) of Brescia, Italy. Informed written consent was obtained from all participants or their care providers. Participation was voluntary, and the data were processed anonymously.

### Face-n-Food task

The Face-n-Food task was administered to participants. The task is described in detail elsewhere^61^. In brief, for this task, all participants were presented with a set of images, one by one, in the predetermined order from the least to most resembling a face (images 1 to 10). This order was determined in the previous study with TD volunteers^61^. This fixed order had been used, because once seen as a face, Face-n-Food images are often processed with a strong face-dominating bias. On each trial, participants had to perform a spontaneous recognition task: they were asked to briefly describe what they saw. Their reports were recorded, and then analysed by independent experts. For further data processing, the responses were coded as either non-face (0) or face (1) report. No immediate feedback was provided. To avoid time pressure that can potentially cause stress and negative emotional and physiological reactions blocking cognitive processes, there was no time limit on the task. With each participant, the testing procedure lasted no longer than 10–20 min.

### Neuropsychological assessment

Several additional tasks directed at assessment of visuospatial and cognitive abilities were routinely administered to PB individuals. All of them represent well-established tools for neuropsychological assessment^54,70^: (1) The Block Construction test (which is a subtest of the test battery NEPSY-II^126^, adapted to Italian population^127^) is designed to assess the visuospatial and visuomotor ability. Participants are asked to reproduce three-dimensional constructions from models or from two-dimensional drawings under time limit. (2) The Visual Motor Integration test (VMI^128^, adapted to Italian population^129^), serves for obtaining visual-motor integration quotient (VMIQ). Patients are first shown a set of progressively complex geometric shapes (in total 27), and on each item they are asked to draw it by him/herself. (3) In the Street Completion Test^129,130^, participants have to recognize 11 black-and-white fragmented images that represent real objects with increasing ambiguity. (4) The Unusual Perspective test and Unusual Lighting test developed from scratch^130,131^ contain 22 colour photographs of objects (22 images of objects photographed from unusual viewpoints and 22 images of objects presented under unusual lighting conditions) and photographs of the same objects presented in a conventional manner. Participants are asked to recognize each object. (5) The Imagery Figure (IF) test contains 20 out of the 128 items of the Birmingham Object Recognition Battery^131^, defined as *easy* in the original battery, and seemed appealing to children. Ten of them represent depictions of objects and the rest ten are imaginary fanciful images combining features of two objects or animals (e.g., a camel with a goose’s head). Participants have to indicate whether each item represents an animal or object that really exists. (6) In addition, two tests (described below) are aimed at assessment of semantic categorization^129,130^. Both the Matching by Class and the Matching by Function tests consist of 20 out of the 64 items of the original test (the Birmingham Object Recognition Battery^131^). In ten out of 20 selected items, participants are asked to match objects belonging to the same class, and in the rest to match objects that are functionally related. Each item consists of the target image and two images, one that matches the target and a distractor. For each participant on each test, z-scores were computed by using the following formula: ‘raw score’ minus ‘average score’ (for participants of this age) divided by SD (for participants of this age).

### Data analysis

At outset, all data sets were routinely analysed for normality of distribution by using Shapiro–Wilk tests with subsequent usage of either parametric (for normally distributed data) or, otherwise, non-parametric statistics. For not normally distributed data sets, additionally to means and SDs, Mdns and 95% CIs are reported throughout the text.
